# Endoglin Requirement for BMP9 Signaling in Endothelial Cells Reveals New Mechanism of Action for Selective Anti-Endoglin Antibodies

**DOI:** 10.1371/journal.pone.0050920

**Published:** 2012-12-27

**Authors:** Olivier Nolan-Stevaux, Wendy Zhong, Stacey Culp, Kathy Shaffer, Joseph Hoover, Dineli Wickramasinghe, Astrid Ruefli-Brasse

**Affiliations:** 1 Oncology Research, Amgen, South San Francisco, California, United States of America; 2 Therapeutic Discovery, Amgen, Seattle, Washington, United States of America; Centro Cardiologico Monzino, Italy

## Abstract

Endoglin (ENG), a co-receptor for several TGFβ-family cytokines, is expressed in dividing endothelial cells alongside ALK1, the *ACVRL1* gene product. *ENG* and *ACVRL1* are both required for angiogenesis and mutations in either gene are associated with Hereditary Hemorrhagic Telangectasia, a rare genetic vascular disorder. ENG and ALK1 function in the same genetic pathway but the relative contribution of TGFβ and BMP9 to SMAD1/5/8 activation and the requirement of ENG as a co-mediator of SMAD phosphorylation in endothelial cells remain debated. Here, we show that BMP9 and TGFβ1 induce distinct SMAD phosphorylation responses in primary human endothelial cells and that, unlike BMP9, TGFβ only induces SMAD1/5/8 phosphorylation in a subset of immortalized mouse endothelial cell lines, but not in primary human endothelial cells. We also demonstrate, using siRNA depletion of *ENG* and novel anti-ENG antibodies, that *ENG* is required for BMP9/pSMAD1 signaling in all human and mouse endothelial cells tested. Finally, anti-ENG antibodies that interfere with BMP9/pSMAD1 signaling, but not with TGFβ1/pSMAD3 signaling, also decrease *in vitro* HUVEC endothelial tube formation and inhibit BMP9 binding to recombinant ENG *in vitro*. Our data demonstrate that BMP9 signaling inhibition is a key and previously unreported mechanism of action of TRC105, an anti-angiogenic anti-Endoglin antibody currently evaluated in clinical trials.

## Introduction

Endoglin (ENG), a dimeric membrane glycoprotein also known as CD105, is differentially expressed at the surface of hypoxic or dividing endothelial cells (EC) and is expressed in the endothelium of virtually all solid tumors [1], [2]. ENG can associate with a number of Type I and Type II TGFβ receptors and is defined as a Type III TGFβ co-receptor based on its homology to TGFBR3 (a.k.a. betaglycan) and its ability to bind diverse TGFβ-family cytokines including Activin, TGFβ1, BMP2, BMP7 and BMP9/10, in vitro [3] or in cell culture assays [4].

Multiple lines of evidence indicate that the key angiogenic function of ENG is linked to the function of the ALK1 receptor, the product of the *Activin-like Receptor Kinase 1* gene (*ACVRL1*) also preferentially expressed in EC [5], [6]. First, most individuals affected with Hereditary Hemorrhagic Telangectasia (HHT), an autosomal-dominant genetic disorder leading to severe vascular malformations, carry heterozygous mutations in either *ENG* (HHT type 1) or *ACVRL1* (HHT type 2) [7]. Second, homozygous *Eng* and *Alk1* mutant mouse embryos closely phenocopy each other and die at day 11.5 from severe vascular malformations attributed to defective angiogenesis [8]. In addition, ALK1 binds to ENG [9] and phosphorylates ENG in its cytoplasmic domain [10]. Thus ALK1 and ENG function in the same genetic and biochemical pathways and result in the phosphorylation and activation of the SMAD1/5/8 sub-family of transcription factors in EC [11], [12].

While genetic evidence clearly identifies ENG and ALK1 as co-receptors required for angiogenesis and vascular homeostasis, the ligand involved in mediating these endothelial functions is less well defined. TGFβ and BMP9 have both been proposed to be the key cytokine upstream of ALK1/pSMAD1/5/8 signaling in EC. Several observations support the TGFβ hypothesis: first, early studies showed that ENG is associated with TGFβ receptor type II in primary EC [13], [14]; second, TGFβ was shown to trigger SMAD1/5/8 phosphorylation in mouse embryonic endothelial cells (MEEC) and bovine aortic endothelial cells (BAEC) [15]; and third, *ENG* is required for TGFβ/ALK1-mediated phosphorylation of SMAD1/5/8 in MEECs [16]. These observations suggested a model whereby HHT is a TGFβ-dependent disease [17]–[9].

More recent evidence, however, indicates that BMP9 and BMP10, two highly related members of the Bone Morphogenetic Protein family, are the key cytokines upstream of ALK1/pSMAD1/5/8 signal transduction in EC. BMP9 and 10 were shown to induce ALK1-dependent SMAD1/5/8 phosphorylation in primary EC [12], [20]. BMP9 and BMP10 appear to be the cognate ligands of ALK1 since BMP9 only associates with ALK1 and no other ALK receptor [21], and ALK1 only binds to BMP9 and BMP10 but not to TGFβ1-3 or any other of the 26 ligands of the TGFβ family [22]. In addition, BMP9 is the factor in human plasma responsible for serum/plasma-induced SMAD1/5/8 phosphorylation in human micro-vascular endothelial cells from the dermis (HMVECd), a primary EC type [23]. Taken together, these observations support a model where HHT results from a deficit in BMP9-10 signaling [7]. However, a recent study showing that BMP9 and TGFβ cooperate to induce EC proliferation while antagonizing each other at the level of SMAD1/5/8 activation [24] illustrates the ongoing and still unresolved debate surrounding the relative importance of TGFβ and BMP9 to SMAD1/5/8 activation in EC [7], [25]–[27].

Existing studies are also at odds regarding the importance and requirement of ENG for ALK1/pSMAD1/5/8 signaling in EC. For example, while ENG was shown to potentiate ALK1/pSMAD1 signaling in EC [12] and to be required for TGFβ/SMAD1/5/8 signaling in MEECS [16], a more recent study showed that ENG depletion, using an *ENG* siRNA, did not affect BMP9/SMAD1/5/8 signaling in human pulmonary endothelial cells (HPAEC) [28].

To better understand the mechanism of ENG inhibition that elicits an anti-angiogenic response, we analyzed the requirement of ENG for endothelial SMAD activation. Using primary human ECs, we demonstrate that SMAD1/5/8 phosphorylation is achieved through BMP9, not TGFβ signaling, and that TGFβ induces a parallel, ENG-dependent, “canonical” SMAD2/3 phosphorylation response in ECs. We also show, using siRNA and selective ENG-neutralizing antibodies that ENG is required for optimal BMP9 signal transduction in all human and mouse ECs tested. Finally, we find that these ENG-neutralizing antibodies induce profound defects in endothelial tube formation *in vitro*, thus identifying BMP9 signaling inhibition as a previously unreported mechanism of action of potentially therapeutic anti-angiogenic anti-ENG antibodies.

## Materials and Methods

### Ethics statement

Rats were cared for in accordance to the *Guide for the Care and Use of Laboratory Animals, 8^th^* Edition from the National Institute of Health. Animals were housed at a facility internationally-accredited by the Association for Assessment and Accreditation of Laboratory Animal Care (AAALAC), in ventilated micro-isolator housing. Animals had ad libitum access to feed and water via automatic watering system. Animals were maintained on a 12 hr:12 hr light:dark cycle, in rooms at 22°C and 45% humidity. Our research protocol and animal housing plan were approved by the Amgen Washington Institutional Animal Care and Use Committee (Amgen Washington IACUC, Protocol #2009-00152).

### Cell culture

The following human primary ECs were used and cultured in the vendor's recommended media: Human Umbilical Vein Endothelial Cells (HUVEC – Lonza #C2517A) grown in EGM® (Lonza, cat# CC-3124), Human Microvascular Endothelial Cells adult dermis (HMVECd – Invitrogen #C0115C) grown in Medium 131 supplemented with MVGS (Gibco/Cascade Biologics #M-131-500), Human Aortic Endothelial Cells (HAEC – Invitrogen #C0065C) and Human Pulmonary Arterial Endothelial Cells (HPAEC – Invitrogen #C0085C) grown in Medium 200 supplemented with LSGS kits (Gibco/Cascade Biologics #M-200-500 and S-003-K). The following mouse EC lines were acquired from ATCC and cultured in the recommended media: MS-1 (ATCC# CRL-2279), EOMA (ATCC# CRL-2586), bEnd.3 (ATCC# CRL-2299), SVR (ATCC# CRL-2280), C166 (ATCC# CRL-2581).

### pSMAD1/SMAD1 detection assay

ECs were stimulated with recombinant human BMP9 (R&D systems #3209-BP-010/CF) or recombinant TGF-beta 1 (R&D systems #100-B-010/CF) at the indicated concentration following 3 hours of serum starvation. A Meso Scale Discovery (MSD) capture Elisa assay was developed to detect phosphorylated SMAD1 and total SMAD1 in protein extracts. Fifty high density 96-well plates were custom-coated by MSD using 500 μg of a monoclonal anti-SMAD1 antibody (Santa Cruz #sc-81378). Two different detection antibodies were used to detect phosphorylated SMAD1/5/8 (Cell Signaling Technology #9511) and total SMAD1 (Cell Signaling Technology #9743). Detection was conducted using the recommended MSD's protocol and buffers. In brief, HUVECs were lifted with Accutase (Invitrogen #A1110501), washed 2x in PBS and plated in a 96-well plate at 2500 cells/well in 25 μl of EBM medium (Lonza #CC-3121). After 3 hours of serum starvation, antibodies were added to the wells in 25 μl of EBM. After 1 hour, 2.5 μl of BMP9 (2 ng/ml) was added to each well. After 30 min, cells were lyzed with 50 μl of MSD lysis buffer 2x (150 mM NaCl, 20 mM Tris, pH 7.5, 1 mM EDTA, 1 mM EGTA, 1% Triton X-100, supplemented with phosphatase and protease inhibitors) and kept frozen at −20°C. 40 μl of lysate were used for the MSD assay (manufacturer's instructions). MSD plates were washed on an ELx405 Select CW plate washer (from Biotek) and the signal was read on a Sector Imager 6000 (from Meso Scale Discovery).

### siRNA assay

siRNAs against ENG and control siRNAs were ordered from Qiagen and reversed-transfected into HUVECs at a concentration of 30 nM using 0.3 μl of Lipofectamine™ RNAiMax (Invitrogen #13778-100) for each well of a 96-well plate. The following siRNAs were used: Rand1-AACGCAGAGTTCGACCGTTTA, Rand2-AAGGGCAACATCAAGGTTTAT, ENG1-CTGTCTGGTTGCACAAGCAAA, ENG2-CCCACTGCACTTGGCC-TACAA, ENG3-ACCAATAAATCAGACCATGAA, ENG4-ACCCAAGTCCCTGTCATTTGA, ENG5-CTCGGAG-AGCAGCAGCACCAA, ENG6-CTGGGATATGGCTGCCCAGGA and ENG7-ACCCTGGGAGCCAGTCCTCCA. Viability assays were performed 72 hours after transfection and protein extracts for western blots were generated 48 hours after transfection.

### Antibodies and Western Blots

The following primary antibodies were used for western blot detection: ENG (anti-human CD105, BD Biosciences, Cat# 611315), pSMAD1/5/8 (Cell Signaling Technology #9511), SMAD1 (R&D Systems, Cat# AF2039), SMAD3 (Abcam #ab75512), pSMAD3 (Abcam #ab51451), beta-Actin (Sigma #A2228). The following antibodies were used in functional assays: MAB3209 (neutralizing anti-human BMP9 monoclonal IgG2b antibody – R&D Systems #3209), M999 (monoclonal Rat anti-Human ENG Ab developed at Amgen), cSN6j (chimeric human IgG2 derived from the SN6j anti-human ENG monoclonal IgG1k antibody [29] – see below for detailed description), TRC105 (chimeric human IgG1 derived from the SN6j anti-human ENG monoclonal IgG1k antibody [29] – see below for detailed description), M1041, M1042 and M1043 (monoclonal Rat anti-mouse Eng Ab developed at Amgen), SN6h (mouse monoclonal IgG1k anti-ENG Ab – ThermoFisher Scientific #MS-1290-P1ABX – NaN_3_ and BSA free), and SN6 (mouse monoclonal IgG1k anti-ENG Ab – eBioscience #16-1057 – NaN_3_ and BSA free). Isotype control antibodies were obtained from various sources: Functional Grade mIgG1k (mouse IgG1kappa – eBioscience #16-4714), mIgG2b (mouse IgG2b – R&D Systems #MAB004), rIgG2a (rat IgG2a – R&D Systems #MAB006), hIgG1 (human anti<KLH> IgG1 – Amgen), hIgG2 (human anti<KLH> IgG2 – Amgen).

The chimeric cSN6j and TRC105 antibodies were produced at Amgen in a human IgG_2_ and IgG1 scaffold, respectively. The published amino acid sequences of the V_H_ and V_L_ domains from the SN6j mouse antibody [29] were cloned in human IgG_2_ or IgG1 expression vectors and recombinant antibodies were purified following transient transfection of mammalian cells. Antibodies M999, M1041, M1042 and M1043 were generated using traditional hybridoma techniques. Briefly, two sets of Lewis rats were immunized with either human or mouse Endoglin in both soluble and cell expressed form. Spleen cells were harvested and fused by electrofusion to either 653 or Sp2/0 myeloma cells.

The following fluorescently-labeled secondary antibodies from Li-Cor were used: IRDye 800CW goat anti-mouse (#926-32210), IRDye 800CW goat anti-rabbit (#926-32211), IRDye 800CW donkey anti-goat (#926-32214), IRDye 680 goat anti-mouse (#926-32220), IRDye 680 goat anti-rabbit (#926-32221), IRDye 680 donkey anti-goat (#926-32224). Western blots images were obtained on an Odyssey® imager (Li-Cor Biosciences).

### *In vitro* cell growth assay

HUVECs were plated on 96-well plates on day 0 (1000 cells per well) in EGM® media (Lonza #CC-3124) with 0.1% FBS and supplemented with test antibodies. After 72 hours, cell growth was assessed using Cell Titer Glo® assay (Promega #G7570).

### *In vitro* tube formation assay

CellPlayer™ Angiogenesis Livekits (Essen Bioscience #4436) were used to monitor the effect of anti-BMP9 and anti-Endoglin antibodies on *in vitro* endothelial tube formation. GFP-labeled HUVEC tube formation was monitored using an Incucyte imaging system (Essen BioScience). Manufacturer's protocol was adhered to strictly. In brief, treatment with PBS, Suramin (20 μM), or antibodies (10–20 μg/ml) was initiated 4 days after cell plating, media was replaced on days 4, 5, 7, 10 and 12 after cell plating and tube length or branch points were measured daily using Incucyte. Data for day 13.5 post-plating are shown.

### Quantitative PCR assay

Total RNA from HUVECs was prepared using the RNAeasy Mini kit (Qiagen) following the manufacturer's recommendations. cDNA synthesis was performed using iScript (Bio-Rad). PCRs were performed using the following TaqMan® assays (Applied Biosystems): Hs00195432_m1 (*SMAD1*), Hs00183425_m1 (*SMAD2*), Hs00969210_m1 (*SMAD3*), Hs00195437_m1 (*SMAD5*), Hs00195441_m1 (*SMAD8*), Hs99999903_m1 (*ACTB*), Hs02758991_g1 (*GAPDH*), Hs00178579_m1 (*SMAD6*) and Hs01126607_g1 (*SERPINE1*). Each assay was tested for efficiency >0.8. Quantitative PCR reactions were performed on an ABI7900HT Sequence Detection System. Ct values were determined and subtracted to obtain the ΔCt [ΔCt  =  Ct (test locus) − Ct (control locus)]. Relative fold difference was calculated as 2^−ΔCt^×100.

### Bio-Layer Interferometry

#### M999 competition assay

Streptavidin biosensors (ForteBio, #18-5019) were hydrated in Sample Diluent (ForteBio #18-5028) for 10 min, and then loaded onto the OctetRED. Biotinylated recombinant human Endoglin (Amgen Lot: 107364.38) was loaded to saturation after a 60 sec. baseline in sample diluents. The baseline was re-established for 60 sec and 10 μg/mL of M999 or a mouse IgG1k control (eBiosciences, #16-4714-82) were loaded for 300 sec. The sensors were immediately moved to 10 μg/mL of the test antibodies: M999, TRC105, SN6 (eBiosciences, # 16-1057-82), SN6h (Neomarker, #MS-1290-P1ABX) or IgG1k control and binding was observed for 300 sec. All sensorgrams were aligned to the last 5 sec of the baseline, and reporter points at 290 and 590 sec were subtracted to give the Wavelength Shift values.

#### BMP-9 competition assay

Streptavidin biosensors (ForteBio, #18-5019) were hydrated in Sample Diluent (ForteBio #18-5028) for 10 min, and then loaded onto the OctetRED. Biotinylated recombinant human Endoglin (Amgen Lot: 107364.38) was loaded to saturation after a 60 sec baseline in sample diluents. The baseline was reestablished for 60 sec and 10 μg/mL of the test antibodies: M999, TRC105, SN6 (eBiosciences, # 16-1057-82), SN6h (Neomarker, #MS-1290-P1ABX) and the IgG1k control were loaded for 300 sec. The sensors were immediately moved to 10 μg/mL recombinant human BMP9 (R & D Systems, #3209-BP/CF) and binding was observed for 300 sec. All sensorgrams were aligned to the last 5 sec of the baseline, and reporter points at 290 and 590 sec were subtracted to give the Wavelength Shift values.

## Results

### BMP9, but not TGFβ1, induces SMAD1/5/8 phosphorylation in primary human EC

**Figure 1:**
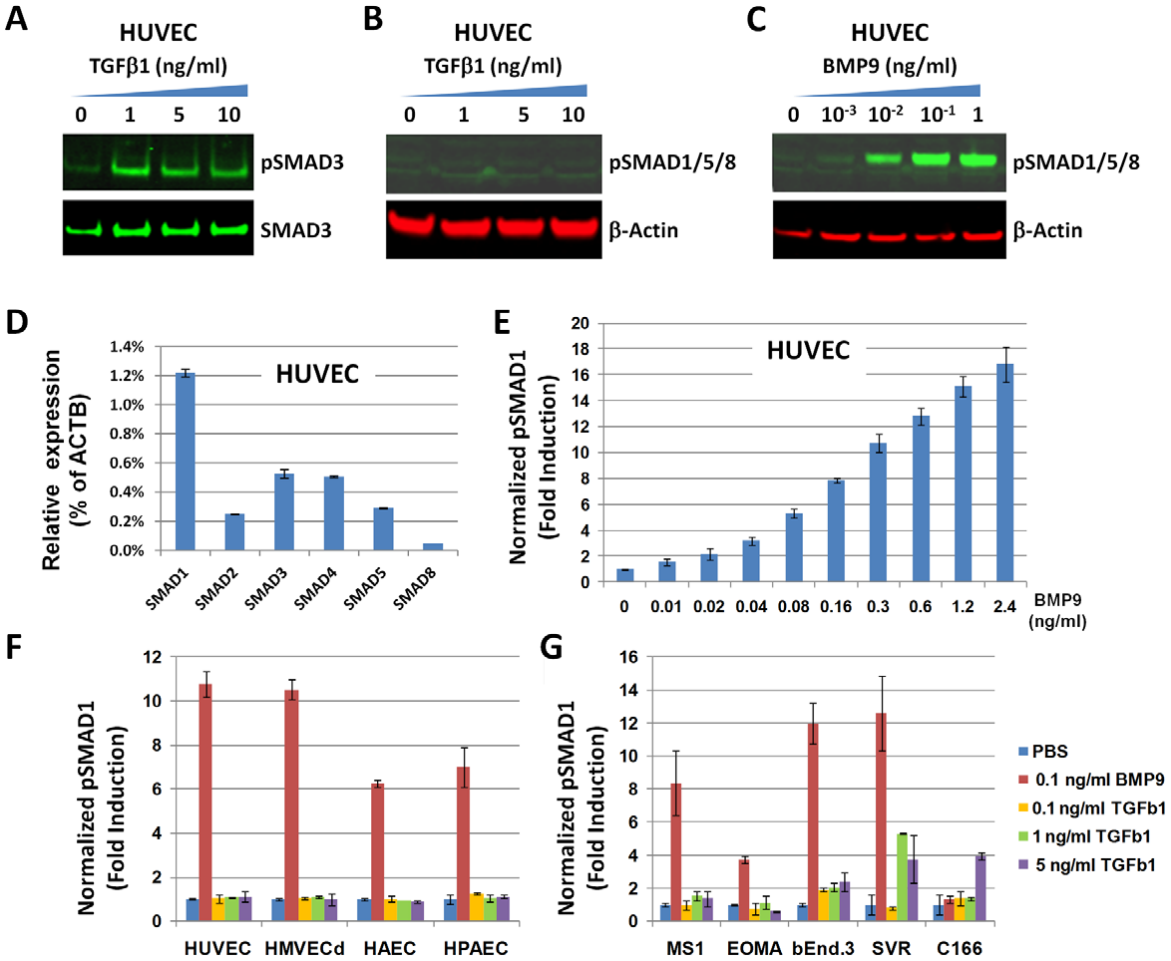
The SMAD1/5/8 pathway in primary endothelial cells is activated by BMP9, not TGFβ1. A–C) HUVECs were serum-starved for 3 hours and stimulated with increasing amounts of TGFβ1 or BMP9 for 30 minutes. Levels of SMAD3, pSMAD3, pSMAD1/5/8 and β-Actin in the total cell extracts were assessed by western blotting. (D) Total RNA extract from HUVECs was subjected to reverse transcription and quantitative PCR assessment of the SMAD1, 2, 3, 5 and 8 transcripts relative to the levels of the β-Actin transcript. (E) HUVECs were serum-starved for 3 hours and treated with increasing amounts of BMP9 and levels of phospho-SMAD1 normalized for levels of total SMAD1 were monitored using a SMAD1/pSMAD1 MSD assay. (F-G) Primary human ECs (HUVEC, HMVECd, HAEC, HPAEC –2500 cells/well) and immortalized mouse EC lines (MS1, EOMA, bEnd.3, SVR, C166–1250 cells/well) were serum-starved for 3 hours and monitored for pSMAD1 induction with a SMAD1/pSMAD1 MSD assay upon treatment with BMP9 or increasing amounts of TGFβ1 for 30 minutes. Results are the mean +/− standard deviation of technical triplicates.

To understand the role of ENG in endothelial cell signaling, we first tested the ability of TGFβ1 and BMP9 to induce SMAD phosphorylation in primary human ECs. First, we stimulated HUVECs with increasing levels of TGFβ1 or BMP9 following serum-starvation, to minimize base-line SMAD1/5/8 and SMAD3 phosphorylation detected in the presence of serum. Whereas TGFβ1 induced SMAD3 phosphorylation in HUVECs (Fig. 1A), it was not capable of triggering SMAD1/5/8 phosphorylation (Fig. 1B). BMP9, however, elicited a SMAD1/5/8 phosphorylation response in HUVECs (Fig. 1C), indicating that the non-canonical “cross-over” TGFβ1/pSMAD1/5/8 pathway [2] previously described in MEECS and BAEC [15] was not operative in this primary EC type. Using quantitative PCR, we confirmed the up-regulation of the *SMAD6* mRNA upon BMP9 stimulation and of the *SERPINE1*/*PAI-1* mRNA upon TGFβ1 stimulation of HUVECs (Fig. S1), genes previously reported to be strongly induced in endothelial cells by BMP9 [12] or TGFβ [15], respectively. To facilitate the assessment of SMAD1 phosphorylation (the SMAD from the SMAD1/5/8 sub-group whose mRNA is the most abundant in HUVECs by quantitative PCR – Figure 1D), we developed a high throughput MSD assay to detect total and phosphorylated SMAD1 in cellular extracts (see Materials and Methods). We detected a clear dose-dependent BMP9/pSMAD1/5/8 response in HUVECs by western blot (Fig. 1C), as well as a dose-dependent BMP9/pSMAD1 response using our MSD assay (Fig. 1E). Using this assay, we tested the response of four primary human EC types to BMP9 and TGFβ1 (HUVEC, HMVECd, HAEC and HPAEC). Again, we observed a robust BMP9/pSMAD1 but no TGFβ1/pSMAD1 response in each of these primary human EC types (Fig. 1F).

Given that TGFβ/pSMAD1/5/8 signaling was previously described in an immortalized mouse EC type (MEEC) [15], we obtained a number of immortalized mouse EC lines and investigated their response to TGFβ1 or BMP9. All these mouse EC lines, except C166, demonstrated a robust BMP9/pSMAD1 response, and three lines (bEnd.3, SVR and C166) were capable of inducing pSMAD1 modestly in response to TGFβ (Fig. 1G), indicating that TGFβ1/pSMAD1 signaling can be observed in a subset of immortalized EC lines, but is not a feature of primary human ECs.

### ENG is required for BMP9/pSMAD1 signaling in endothelial cells

**Figure 2:**
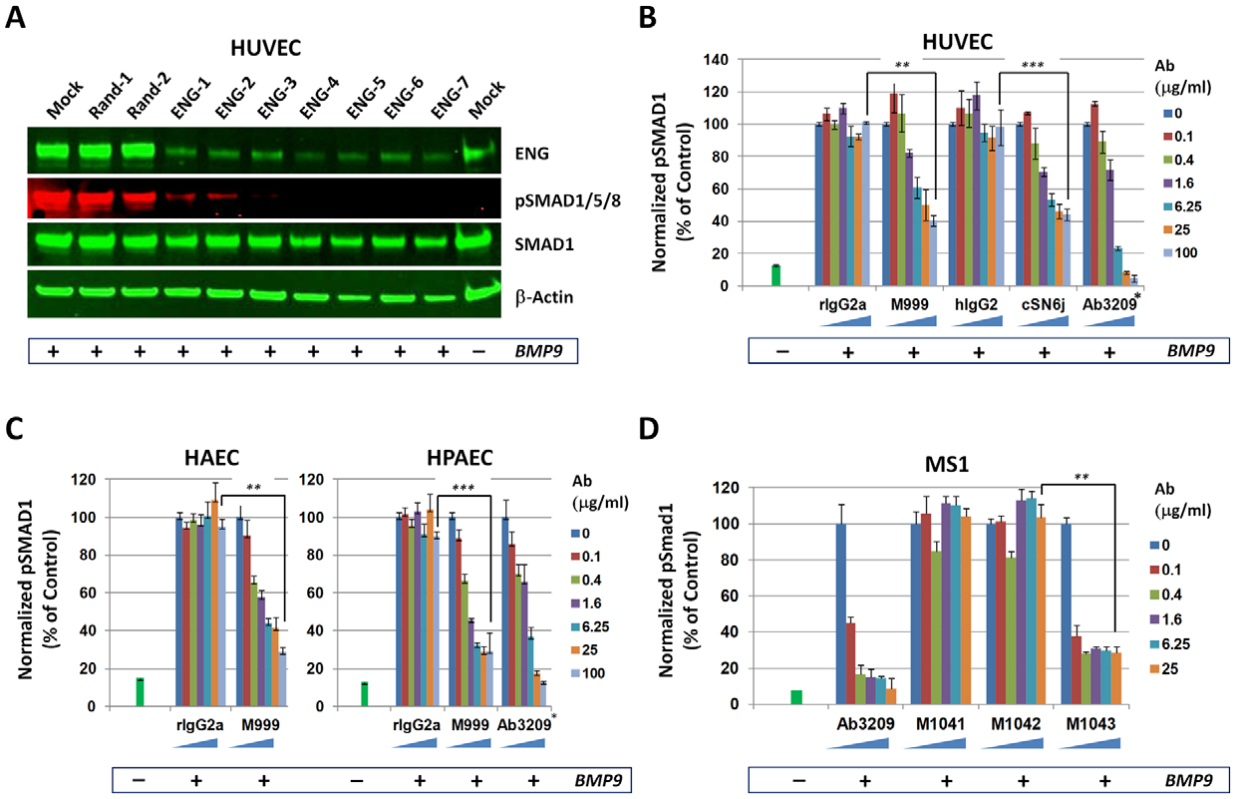
ENG is required for BMP9-mediated SMAD1/5/8 phosphorylation in endothelial cells. A) HUVECs were reversed-transfected in a 96-well plate with Mock, Random or ENG siRNAs for 48 hours then serum-starved for 3 hours prior to stimulation with BMP9 (0.1 ng/ml) (+) or PBS (−). Levels of ENG, pSMAD1/5/8, SMAD1 and β-Actin in the total cell extracts were measured by western blotting. (B–D) ECs were dispensed in each well of a 96-well plate (HUVEC, HPAEC, HAEC –2500 cells/well, MS1–1250 cells/well) in serum free media. Cells were incubated with antibodies at various concentrations for 1 hour and stimulated with BMP9 (0.1 ng/ml) (+) or PBS (−) for 30 min prior to lysis. Levels of pSMAD1 normalized for levels of total SMAD1 were monitored using a SMAD1/pSMAD1 MSD assay. The following antibodies were used: rIgG2a (rat IgG2a isotype control), M999 (rat IgG2a anti-human ENG), hIgG2 (human IgG2 isotype control), cSN6j (human IgG2 anti-human ENG), Ab3209 (mouse IgG2b anti-human BMP9), M1041-43 (rat IgG2a anti-mouse ENG). * Antibody concentrations of Ab3209 used were 10 times lower than other antibodies. Results are the mean +/− standard deviation of technical triplicates (**  =  P Value <10E-04; ***  =  P value <10E-03).

To test the requirement of ENG for the BMP9/pSMAD1 signaling axis observed in HUVECs, we depleted ENG using seven independent siRNA triggers. The ENG-specific siRNAs led to a 70–85% decrease in ENG protein expression compared to the Random siRNA controls, assessed by western blotting (Fig. 2A – ENG lane). When the ENG siRNA-treated HUVECs were stimulated with BMP9, they were greatly impaired in inducing SMAD1/5/8 phosphorylation compared to the Mock and Random siRNA controls (Fig. 2A), demonstrating that ENG is required for BMP9/pSMAD1/5/8 signaling in HUVECs.

We then tested the ability of antibodies raised against human or mouse ENG to block BMP9-induced phosphorylation of SMAD1 in HUVECs and mouse MS1 cells, respectively. We identified 72 human ENG-binding monoclonal antibodies and 230 mouse Eng-binding monoclonal antibodies. Out of these, 4 anti-human ENG antibodies (∼6%) and 16 anti-mouse Eng antibodies (∼7%) met our threshold for BMP9/pSMAD1 signaling inhibition in their respective EC type. We sub-cloned antibody M999, a Rat IgG2a anti-human ENG antibody with the ability to decrease BMP9/pSMAD1 signaling by >60% in HUVECs, HAECs and HPAECs, when pre-incubated for 30 minutes prior to BMP9 stimulation (Fig. 2B–C). The BMP9-blocking activity of M999 was comparable to that of cSN6j, a mouse/human chimeric antibody containing the published V_H_ and V_L_ sequences of the SN6j anti-ENG monoclonal antibody [29] cloned in a human IgG2 antibody backbone (see Materials and Methods). A similar chimeric antibody called TRC105 was derived from the same monoclonal antibody SN6j but in a human IgG1 backbone and was recently tested in a Phase I clinical trial [29]. BMP9/pSMAD1 inhibition by the ENG-binding antibodies was not as pronounced as that of Ab3209, an anti-BMP9 neutralizing antibody serving as positive control, which inhibited BMP9/pSMAD1 signaling by >80% (Fig. 2B–D).

We also sub-cloned a Rat anti-mouse Eng antibody (M1043), which blocked BMP9/pSMAD1 signaling by ∼65% in MS1 cells, unlike two other sub-cloned mouse Eng-binding antibodies of the same isotype (M1041 and M1042), which have no effect on BMP9/pSMAD1 signaling and thus belong to the much larger group of anti-ENG antibodies that bind to their target but have no effect on BMP9 signaling (Fig. 2D). Thus, Endoglin is required for BMP9/pSMAD1 signaling in human and mouse ECs.

### ENG modulates TGFβ1/pSMAD3 signaling in HUVECs

**Figure 3:**
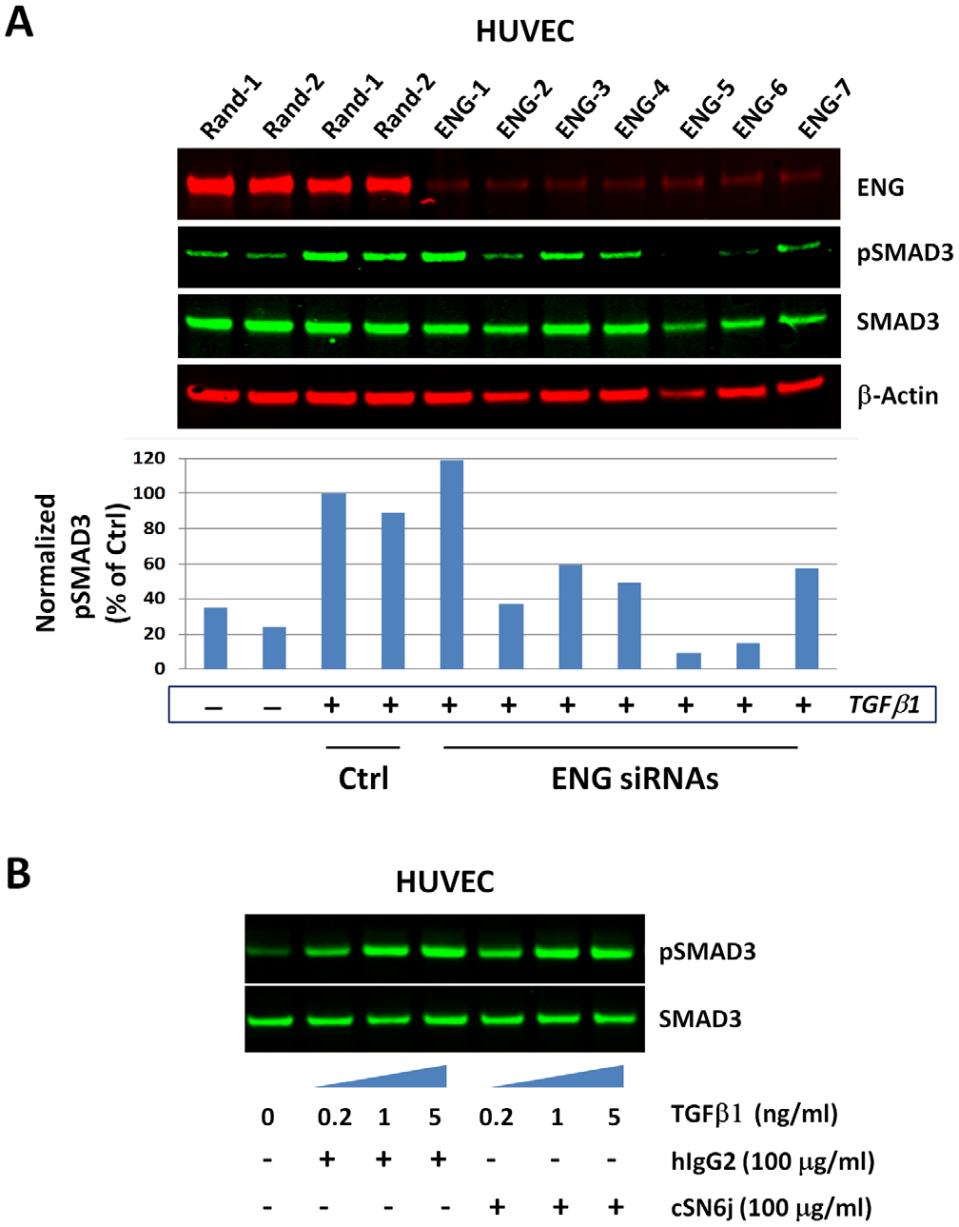
ENG is required for optimal TGFβ-mediated SMAD3 phosphorylation in HUVEC. A) HUVECs were reversed-transfected in a 96-well plate with Random or ENG siRNAs for 48 hours then serum-starved for 3 hours prior to stimulation with TGFβ1 (1 ng/ml) (+) or PBS (−). Levels of ENG, pSMAD3, SMAD3 and β-Actin in the total cell extracts were measured by western blotting and quantified using densitometry analysis of pSMAD3 bands normalized by SMAD3 band levels (graph). (B) HUVECs were dispensed in each well of a 96-well plate (2500 cells/well) in serum free media and starved for 3 hours. Cells were incubated with cSN6j anti-ENG or isotype control hIgG2 antibodies at a concentration of 100 μg/ml (+) for 1 hour and stimulated with increasing amounts of TGFβ1 for 30 min prior to lysis. Levels of pSMAD3 and total SMAD3 were monitored by western blot.

Since TGFβ1 induces a canonical pSMAD3 response in HUVECs (Fig. 1B), we also tested the role of ENG in TGFβ1/pSMAD3 signaling. We pursued SMAD3 as the more abundant SMAD of the SMAD2/3 sub-group, based on qPCR measurement (Fig. 1D). Upon depletion of *ENG* with seven independent ENG siRNAs (Fig. 3A), we stimulated HUVECs with TGFβ1 and measured the resulting SMAD3 phosphorylation response by western blot. TGFβ1/pSMAD3 induction was reduced by 40 to 90% with 6 out of 7 ENG siRNAs used (Fig. 3A), indicating that ENG is also required for “canonical” TGFβ1/pSMAD3 signal transduction in HUVEC. Notably, the anti-ENG cSN6j antibody, which interfered with BMP9/pSMAD1 signaling in HUVEC did not inhibit TGFβ1/pSMAD3 signaling (Fig. 3B), suggesting that different ENG epitopes may be involved in BMP9/pSMAD1 and TGFβ1/pSMAD3 signaling. Thus, ENG is also required for normal TGFβ1/pSMAD3 signaling in HUVECs.

### Anti-ENG antibodies inhibiting BMP9 signaling block HUVEC tube formation

**Figure 4:**
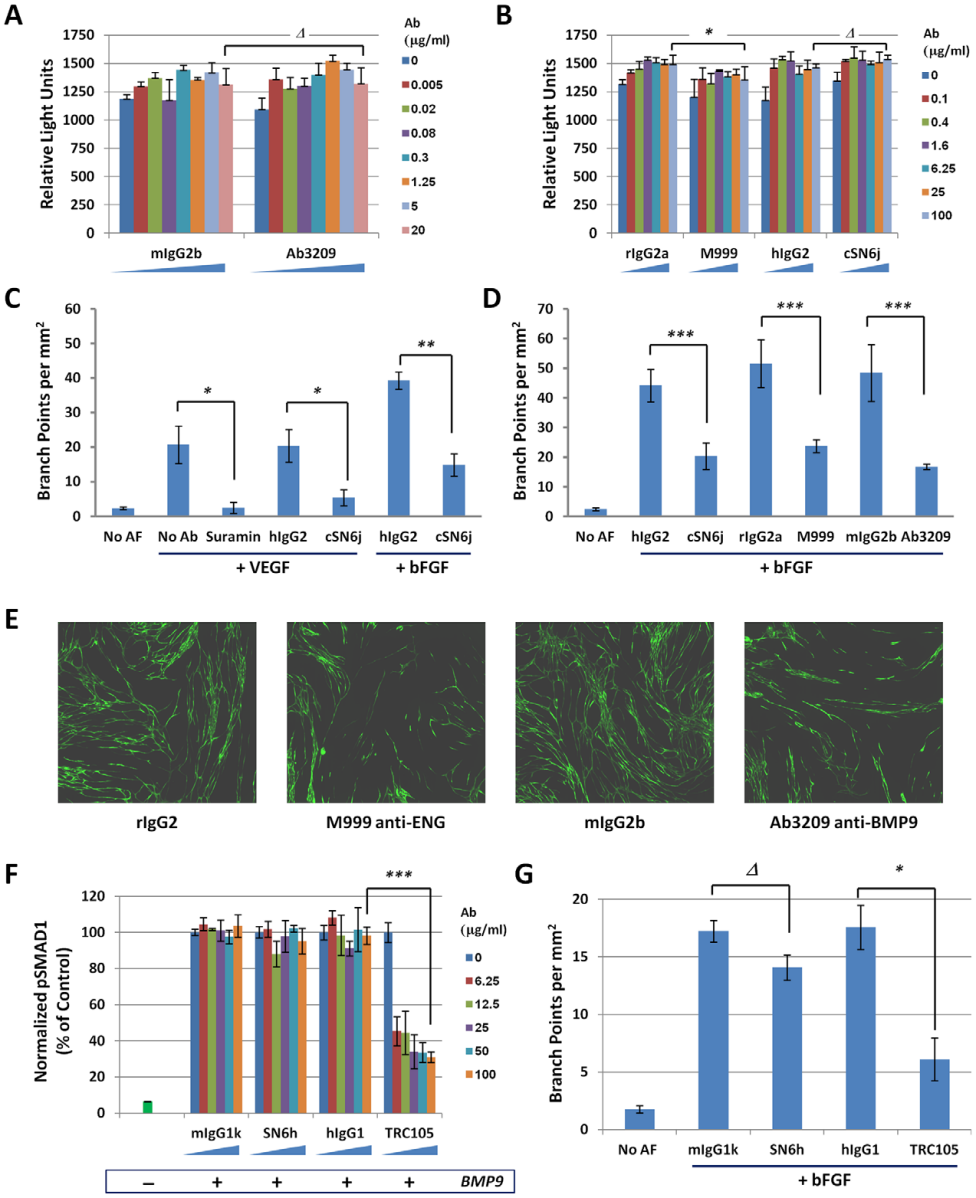
ENG-mediated BMP9 signaling is required for VEGF and bFGF-induced HUVEC tube formation. A–B) HUVECs were dispensed in a 96 well plate (3000 cells/well) and grown in 0.1% FBS and incubated with the following antibodies at various concentrations for 72 hours, with a daily change of media: (A) mIgG2b (mouse IgG2b isotype control) and Ab3209 (mouse IgG2b anti-BMP9); (B) rIgG2a (rat IgG2a isotype control), M999 (rat IgG2a anti-human ENG), hIgG2 (human IgG2 isotype control) and cSN6j (human IgG2 anti-human ENG). After 72 hours, cell growth in each well was assessed using Cell Titer-Glo®. (C–D) GFP-labeled HUVECs admixed with human dermal fibroblasts dispensed in 96 well plates were treated on day 0 with angiogenic factors: VEGF (1 ng/ml) or bFGF (2 ng/ml). Negative controls wells (No AF) contained no angiogenic factors. Measurement of Branch Points were performed at day 13.5 following treatment with PBS (no Ab), Suramin (20 μM – positive control that inhibits RTK-mediated tube formation), or the following antibodies: hIgG2 (isotype control), cSN6j (hIgG2 anti-human ENG), rIgG2a (isotype control), M999 (rIgG2a anti-human ENG), mIgG2 (isotype control) or Ab3209 (mIgG2 anti-human BMP9). Antibody concentrations used in (C): 10 μg/ml; in (D): 20 μg/ml. (E) Representative image of individual wells from the HUVEC tube formation assay following treatment with bFGF (2 ng/ml) and the indicated antibody (20 μg/ml). (F) HUVECs were dispensed in each well of a 96-well plate (2500 cells/well) in serum free media. Cells were incubated with antibodies at various concentrations for 1 hour and stimulated with BMP9 (0.1 ng/ml) (+) or PBS (−) for 30 min prior to lysis. Levels of pSMAD1 normalized for levels of total SMAD1 were monitored using a SMAD1/pSMAD1 MSD assay. The following antibodies were used: mIgG1k (mouse IgG1k isotype control), SN6h (mouse IgG1k anti-human ENG), hIgG1 (human IgG1 isotype control), TRC105 (human IgG1 anti-human ENG). (G) GFP-labeled HUVECs admixed with human dermal fibroblasts dispensed in 96-well plates were treated on day 0 with bFGF (2 ng/ml); No Angiogenic Factor (No AF) wells did not receive bFGF. Measurement of Branch Points were performed at day 13.5 following treatment with the following antibodies: mIgG1k (mouse IgG1k isotype control), SN6h (mouse IgG1k anti-human ENG), hIgG1 (human IgG1 isotype control), TRC105 (human IgG1 anti-human ENG). Antibody concentration used in (G): 10 μg/ml. Results are the mean +/− standard deviation of technical triplicates. Results are the mean +/− standard deviation of technical triplicates (Δ =  P value >0.05; *  =  P value <0.05; **  =  P value <0.005; *** P  =  value <0.001).

We investigated if anti-ENG antibodies that inhibit BMP9/pSMAD1 signaling impacted the normal growth and function of ECs. First, we cultured HUVECs in 0.1% serum medium for 72 hours in the presence of two ENG antibodies that interfere with BMP9 signaling (M999 and cSN6j), and with a BMP9-neutralizing antibody (Ab3209). These antibodies had minimal to no effect on HUVEC cell growth under these conditions (Fig. 4A–B). The only significant difference appeared with M999, which showed a marginal 9% decrease in cell growth over 72 hours.

Second, we assessed the functional significance of BMP9/pSMAD1 signal inhibition by ENG antibodies in an *in vitro* HUVEC tube-formation assay [30]. In this assay, GFP-labeled HUVECs co-cultured with unlabeled human dermal fibroblasts mimic angiogenesis by forming *in vitro* tubular vascular networks in the presence of Fetal Calf Serum and brain extracts, and in response to recombinant VEGF or bFGF. We observed that cSN6j, an anti-ENG antibody that inhibited BMP9 signaling in HUVECs by ∼60% (Fig. 2B), efficiently decreased HUVEC tube formation driven by VEGF (>70% inhibition) or bFGF (>60% inhibition), as measured by the density of Branch Points in the vascular network [31] (Fig. 4C). In this experiment, Suramin, a small molecule blocking multiple Receptor Tyrosine Kinases served as a positive control for tube formation inhibition (Fig. 4C). We also observed that M999, a second BMP9-interfering anti-ENG antibody, and cSN6j had comparable effects on HUVEC tube formation as a BMP9-neutralizing antibody (Ab3209) (Fig. 4D), indicating that BMP9 signal blockade through ENG neutralization phenocopies BMP9 signaling inhibition achieved with a BMP9-neutralizing antibody. The phenotypic effects of the M999 (anti-ENG) and Ab3209 (anti-BMP9) antibodies on endothelial tube formation can be visualized relative to control antibodies (Fig. 4E). Finally, we observed that SN6h, another ENG-binding antibody that did not interfere with BMP9/pSMAD1 signaling (Fig. 4F), did not significantly impact HUVEC tube formation (Fig. 4G), whereas TRC105, a chimeric antibody derived from SN6j and cloned in an IgG1 backbone (similar to the TRC105 antibody currently tested in the clinic) recapitulated the effect of the cSN6j antibody and inhibited both BMP9/pSMAD1 signaling (Fig. 4F) and HUVEC tube formation (Fig. 4G).

Thus, despite having a minimal effect on HUVEC cell proliferation *in vitro*, anti-ENG antibodies that interfere with BMP9/pSMAD1 signaling profoundly impact VEGF- and bFGF-driven *in vitro* HUVEC tube formation, an assay that mimics the interplay between endothelial and peri-vascular fibroblastic cells during angiogenesis.

### M999 and TRC105 compete for ENG binding and inhibit BMP9 binding to ENG

**Figure 5:**
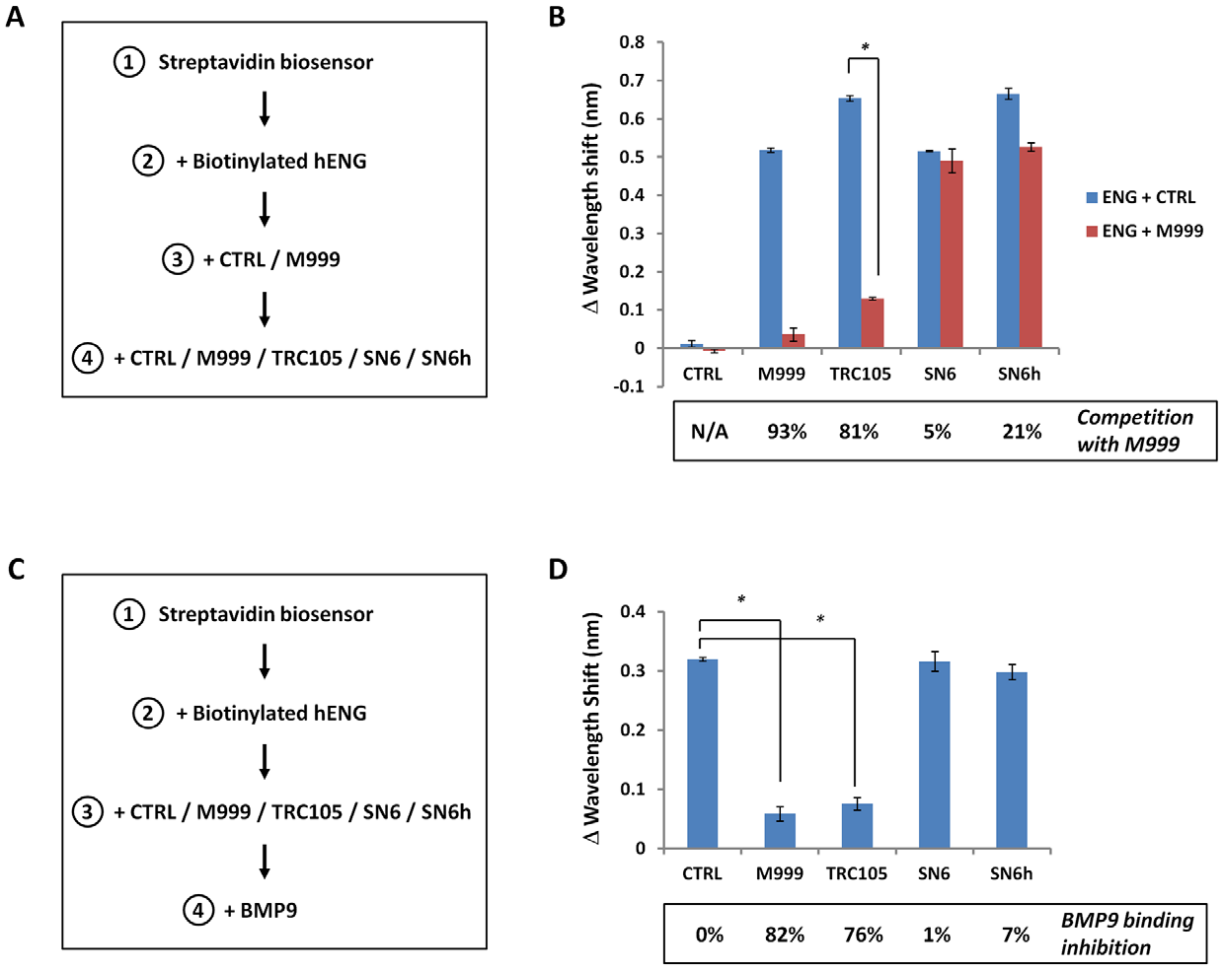
M999 competes with TRC105 for ENG binding and both antibodies block BMP9/ENG interaction. A) Sequence of protein layering in the Bio Layer Interferometry antibody competition assay: 1) a strepatavidin-coated biosensor is loaded to saturation with 2) biotinylated recombinant human Endoglin (10 μg/ml), then with 3) a negative control IgG1k or the M999 anti-Endoglin rat IgG2 antibodies (10 μg/ml) prior to the addition of 4) M999, TRC105, SN6, or SN6h (10 μg/ml). (B) Wavelength shifts (WS) triggered by the addition of the individual anti-ENG antibodies to ENG in the presence of a ctrl antibody (blue bars), or in the presence of the anti-ENG antibody M999 (red bars). Competition with M999, calculated as (WS^CTRL^-WS^M999^)/WS^CTRL^*100 is indicated at the bottom of the graph. (C) Sequence of protein layering in the Bio Layer Interferometry BMP9 binding assay: 1) a strepatavidin-coated biosensor is loaded to saturation with 2) biotinylated recombinant human Endoglin (10 μg/ml), then with 3) a negative control IgG1k or the tested anti-ENG antibodies (M999, TRC105, SN6, SN6h) (10 μg/ml) prior to the addition of 4) BMP9 (10 μg/ml). (B) Wavelength shifts (WS) triggered by the addition of BMP9 in addition to the individual anti-ENG antibodies. BMP9 binding inhibition, calculated as [1-(WS^ENGAB^/WS^CTRLAB^)]*100, is indicated at the bottom of the graph. Results are the mean +/− standard deviation of technical triplicates.

To better understand the binding properties of the different anti-ENG antibodies, we performed an ENG-binding competition assay between M999, TRC105, SN6 and SN6h using Bio Layer Interferometry (BLI) (Fig. 5A). Upon binding of biotinylated recombinant human ENG to a Streptavidin biosensor, we detected a significant binding of all four anti-ENG antibodies to recombinant ENG, compared to a control antibody (Fig. 5B – blue bars). However, prior binding of ENG by M999 significantly decreased additional M999 (by 93%) or TRC105 binding (by 81%) but had a limited impact on additional SN6 or SN6h binding (5% and 21% competition, respectively) (Fig. 5B – red bars), indicating that M999 and TRC105 compete for eptitope binding. BLI curves are presented as additional information (Fig. S2). A reverse experiment, in which TRC105 was first bound to ENG and inhibited additional binding of M999, confirmed the epitope competition (data not shown).

We then used BLI to explore the possible mechanism of BMP9 signaling inhibition observed with TRC105 and M999, but not SN6 and SN6h (Fig. 5C). Upon exposing biotinylated recombinant human ENG to saturating concentrations of all four ENG antibodies (M999, TRC105, SN6 and SN6h) and a negative control antibody, we observed that additional BMP9 binding to recombinant ENG was significantly inhibited by M999 (by 82%) and TRC105 (by 76%), but not by SN6 and SN6h (1% and 7% inhibition respectively) (Fig. 5D). BLI curves are presented as additional information (Fig. S3).

Thus, *in vitro*, M999 and TRC105 compete with each other for binding to their epitope on recombinant ENG and both antibodies significantly inhibit BMP9 binding to ENG.

## Discussion

### BMP9 and TGFβ1 signal in parallel in primary endothelial cells

The relative contribution of BMP9 and TGFβ to SMAD1/5/8 phosphorylation in endothelial cells (EC) remains a matter of debate [2], [32], [33]. Our data strongly indicate that TGFβ1 does not trigger SMAD1/5/8 phosphorylation in primary ECs (Fig. 1). The only primary EC type where TGFβ1/pSMAD1/5/8 has been described to date was bovine aortic endothelial cells (BAEC) [15]; but that result has not be replicated [20], [34]. The EC lines that consistently trigger a TGFβ/pSMAD1/5/8 response are immortalized mouse lines such as MEEC [15] or SVR, bEnd.3 and C166 (Fig. 1). While there is no doubt that TGFβ/pSMAD1 signaling can be detected in many cell types ranging from fibroblasts [35] to cancer cell lines [36], [37], and a variety of immortalized cells of mesenchymal and epithelial origin [38], it does not appear to be a signaling feature of primary human EC. Our findings in this area are in agreement with results from a very recent study [39].

**Figure 6:**
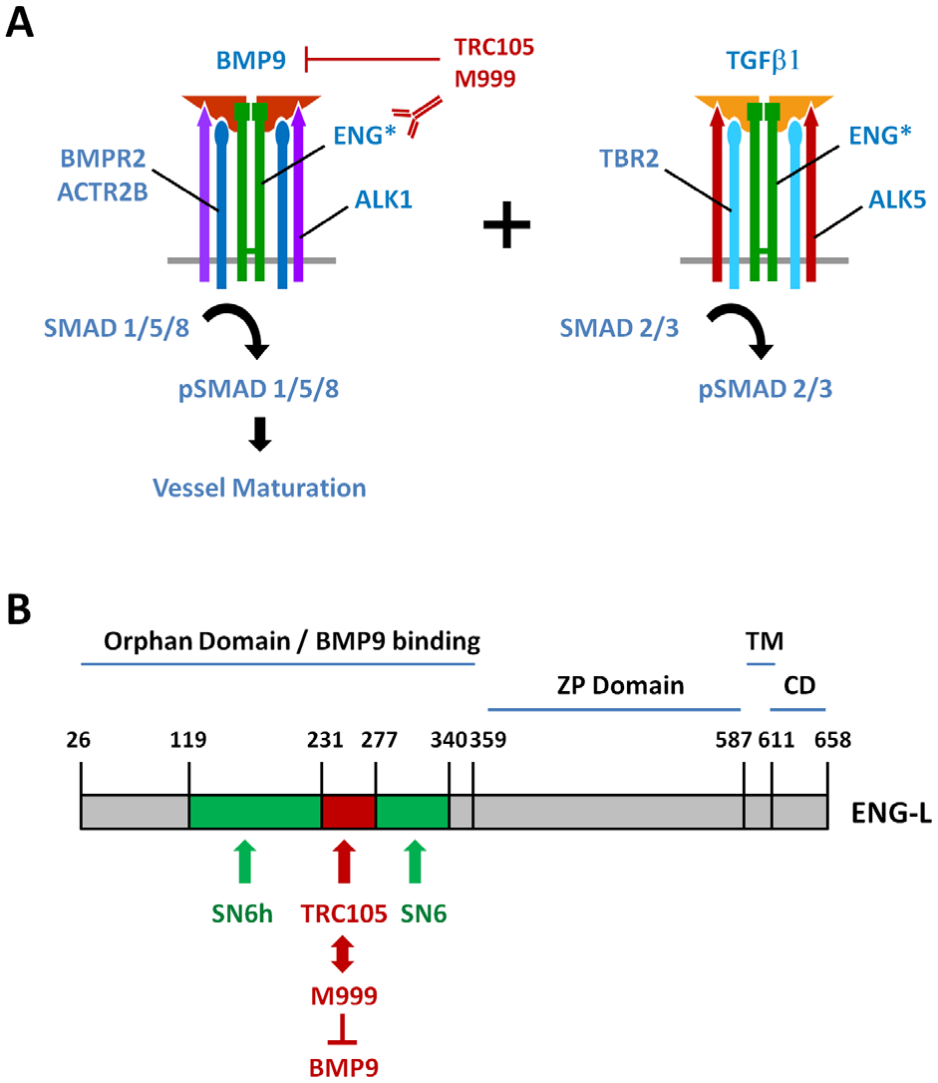
Model of ENG-mediated signaling and BMP9 signaling inhibition by anti-ENG antibodies. A) In primary human ECs, parallel BMP9/ALK1/pSMAD1/5/8 and TFGβ/ALK5/pSMAD2/3 signaling cascades coexist and both pathways demonstrate a requirement for Endoglin (*). Anti-ENG antibodies TRC105 and M999 inhibit BMP9 binding to ENG and BMP9/pSMAD1/5/8 signaling (B) Model of ENG protein with amino acid (AA) numbers depicting: the orphan domain, the ZIP domain (ZP), the trans-membrane domain (TM) and the cytoplasmic domain (CD) [52]. Distinct regions of the orphan domain associate with the following monoclonal antibodies SN6h (AA119-231), TRC105 (AA231-277) or SN6 (AA277-340) [53]. M999, a novel anti-ENG monoclonal antibody competes with TRC105 for ENG binding, indicating that their epitopes overlap. TRC105 and M999 inhibit the interaction between recombinant BMP9 and recombinant ENG.

We also show that BMP9 is required for *in vitro* tube formation (Fig. 4), a result that is consistent with the anti-angiogenic property of soluble ALK1 and soluble ENG, molecules that serve as a sink for BMP9 and BMP10 *in vivo* [3], [22], [26]. In addition, we confirm that TGFβ induces a “canonical” SMAD3 phosphorylation response in primary ECs [40], likely mediated by ALK5, the only cognate Type I TGFβ receptor known to phosphorylate SMAD2 and SMAD3 [41]. Our observations support a model whereby the BMP9/ALK1/pSMAD1/5/8 and the TGFβ/ALK5/pSMAD2/3 axes co-exist in parallel in primary human EC (Fig. 6A).

### Endoglin is required for the modulation of BMP9 and canonical TGFβ signaling in EC

A second area of uncertainty in the field is whether ENG is a required co-mediator of BMP9/pSMAD1/5/8 signaling. ENG is required in MEEC [16] and fibroblasts [35] for TGFβ/pSMAD1/5/8 signaling. ENG is also capable of enhancing BMP9/pSMAD1/5/8 in primary HMVECds [12] and TGFβ/pSMAD1/5/8 in immortalized human HMEC cells [9]. In contrast, another study showed that ENG depletion by a siRNA had no impact on BMP9/pSMAD1/5/8 activation in primary HPAECs [28]. Here, we show that depletion of ENG by seven independent siRNA leads to a dramatic decrease in BMP9/pSMAD1/5/8 output in HUVECs and that a subset of anti-ENG antibodies is capable of inhibiting BMP9/pSMAD1/5/8 signaling in several mouse and human EC types, including HPAEC (Fig. 2). Thus, we provide definitive evidence that ENG is required for optimal BMP9 signaling in all interrogated EC types.

A third area of debate in the field is whether ENG is a required co-mediator of TGFβ/pSMAD2/3 signaling in ECs. In one report, immortalized MEEC expressing reduced levels of ENG displayed decreased SMAD2 phosphorylation in response to TGFβ [15], but in another, immortalized HMEC cells transfected with an ENG siRNA exhibited elevated TGFβ/pSMAD2/3 signaling output [9]. Here, we find that ENG depletion using multiple siRNAs against ENG leads to decreased TGFβ1/pSMAD3 signaling in primary HUVECs (Fig. 3). Our data, obtained in primary HUVECs, support a model in which ENG is a positive co-mediator of canonical TGFβ1/pSMAD3 signaling in normal ECs. Supporting this model, ENG antibodies that inhibit TGFβ/pSMAD2 signaling have been reported [42], but their effect on BMP9 signaling was not explored. A direct comparison of “pure” BMP9-blocking and “pure” TGFβ-blocking ENG antibodies should enable a clearer delineation of the respective contributions of the BMP9/pSMAD1/5/8 and the TGFβ1/pSMAD2/3 signaling cascades to physiological EC function. In addition, to establish if tumor-associated ECs behave like primary endothelial cells with regards to their BMP9 and TGFβ signaling capabilities, a clear next step in the field would be to isolate patient-derived tumor ECs and test their respective pSMAD1/5/8 and pSMAD2/3 outputs in response to BMP9 and TGFβ stimulation.

### BMP9 signaling inhibition is a mechanism of action of anti-angiogenic ENG antibodies

Because of the confusion surrounding the ENG/ALK1 signaling pathway, the signaling mechanism of action of anti-angiogenic ENG antibodies is not well understood [29]. While it is clear that soluble ENG can serve as a sink for BMP9/10 and thus inhibit BMP9 signaling in primary EC [3], it wasn't established if anti-angiogenic ENG antibodies influenced BMP9 signaling. Here, we show for the first time that a small subset of ENG-binding antibodies inhibit BMP9/ALK1/pSMAD1/5/8 signaling and do not affect canonical TGFβ1/ALK5/pSMAD3 signaling in HUVECs.

In addition, we show that the BMP9 signal inhibition obtained with anti-ENG antibodies is sufficient to significantly block *in vitro* HUVEC tube formation to a similar degree as a BMP9-neutralizing antibody (Fig. 4). This result echoes the observation that vascular symptoms of HHT patients are due to *ENG* haplo-insufficiency [43], [44] and that loss of one copy of *Eng* in a genetically engineered mouse model is sufficient to lead to angiogenesis inhibition in a tumor model [45] and to vascular defects reminiscent of HHT [46]. Our data obtained with ENG-neutralizing antibodies reinforce this notion that partial loss of ENG-dependent signaling is sufficient to impinge angiogenesis. Because TRC105, the chimeric anti-ENG antibody based on the SN6j sequence currently in clinical trials, is a human IgG1 antibody, it is capable of eliciting an Antibody Dependent Cell Cytotoxicity (ADCC) response on ENG-expressing ECs [29], and this functionality is thought to contribute to the anti-angiogenic mechanism of action of this antibody [47]. However, our data strongly support a model whereby BMP9 signaling inhibition is also part of the anti-angiogenic mechanism of action of TRC105, since this antibody is capable of blocking BMP9 signaling and vessel formation in an *in vitro* angiogenesis assay (Fig. 4).

The observation that patients treated with TRC105 develop mucosal telangectasias, the hallmark of HHT, in a dose-dependent manner [48] is significant: it demonstrates that ENG-mediated BMP9 inhibition is a likely etiological mechanism underlying HHT; and it provides an easily monitored on-target pharmacodynamic marker of biological response in treated patients. A recent publication demonstrating that BMP9 signaling inhibition is also a mechanism of action of an anti-ALK1 anti-angiogenic antibody currently in clinical trial supports our observation that BMP9 is the key cytokine signaling upstream of the ENG/ALK1/pSMAD1/5/8 pathway [39] in endothelial cells.

### TRC105 and M999 inhibit endogenous BMP9 in tube formation assays

Antibodies M999 and TRC105 phenocopy the anti-BMP9 neutralizing antibody in the HUVEC tube formation assay, suggesting that endogenous levels of BMP9 in the media used in the tubulogenesis assay is sufficient to drive tube formation in the absence of extrinsic BMP9. In our *in vitro* assays detecting pSMAD1/5/8 upon BMP9 stimulation, the cells were always starved and therefore showed very little endogenous pSMAD1/5/8 signal (Fig. 1). However, FCS on its own is capable of inducing SMAD1/5/8 phosphorylation (Fig. S4). Several studies have demonstrated that endogenous BMP9 mediates most of the SMAD1/5/8 phosphorylation activity detected in serum and plasma: BMP9 concentration was measured to be ∼7.5 ng/ml in human serum [23] and ∼2 ng/ml in mouse plasma [49]. In addition, FCS [39] and rat serum [49] were shown to contain endogenous activity triggering SMAD1/5/8 phosphorylation in HUVEC and in rat aortic cells, respectively, which could be neutralized in each case with the same anti-BMP9 antibody used in this study. We conclude that in the tubulogenesis assays presented here, just as in a previously-described HUVEC sprouting assay [39], endogenous BMP9 contained in the FCS used at 2% concentration is sufficient to promote HUVEC tube formation and is inhibited by ENG or BMP9 antibodies.

We also note that the clear inhibition of endothelial tube formation with antibodies M999 and TRC105 occurs in the context of a media containing FBS, indicating that these ENG targeting antibodies can disrupt endothelial cell function in the presence of physiological levels TGFβ1. Based on the levels of TGFβ1 found in normal human serum (40 to 180 ng/ml) [50], we extrapolate that tube formation occurring in 2% FBS happens in the presence of 0.8 to 3.6 ng/ml of TGFβ1, a concentration comparable to the bio-active concentration of TGFβ1 found in normal human plasma, reported to range from 1.7 to 3.2 ng/ml [50]. Our study demonstrates that endogenous TGFβ1 found in FCS is not capable of preventing endothelial tube formation disruption by anti-ENG antibodies.

### Binding properties of TRC105 and inhibition of BMP9 binding to recombinant ENG

Our data indicate that a distinct region of the ENG orphan domain is involved in BMP9 signaling inhibition but does not affect TGFβ signaling. The ENG orphan domain is defined as the portion of the protein located between amino acids E26 and I359 [51], which binds to BMP9 [3], [52]. The binding regions of SN6h, SN6j and SN6 have been previously mapped to the orphan domain: SN6h binds between A119 and G230, SN6j/TRC105 between P231 and E276 and SN6 between Y277 and P338 [53] (Fig. 6B). Since TRC105 and M999 compete with each other for ENG binding, they likely recognize overlapping epitopes (Fig. 5B). Moreover, among the four anti-ENG antibodies tested, only M999 and TRC105 inhibit BMP9/pSMAD1 signaling in EC (Fig. 2B and Fig. 4F), whereas SN6 and SN6h do not (Fig. 4F and Fig. S5). In addition, the blocking of BMP9 signaling by TRC105 and M999 is directly correlated with their ability to inhibit the binding of BMP9 to recombinant ENG (Fig. 5D). Thus, the P231-E276 region of the orphan domain of ENG appears to be structurally important for BMP9 binding to ENG (Fig. 6B).

In conclusion, our work clearly outlines the requirement for ENG in mediating parallel BMP9/ALK1/pSMAD1/5/8 and TGFβ1/ALK5/pSMAD3 signaling pathways in primary human endothelial cells and implicates BMP9 signaling inhibition as a mechanism of action of a subset of anti-ENG antibodies, including M999, a novel anti-ENG antibody and TRC105, currently in clinical development.

## Supporting Information

Figure S1 **Induction of** ***SERPINE1*** **and** ***SMAD6*** **by TGFβ1 and BMP9, respectively.** HUVECs were dispensed in each well of a 96-well plate (2500 cells/well), serum-starved overnight and stimulated with increasing amounts of recombinant BMP9 or TGFβ1. Total RNA extracts were subjected to reverse transcription and quantitative PCR assessment of the *SMAD6* and *SERPINE1* (A.k.a *PAI-1*) transcripts relative to the levels of the GAPDH transcript. Fold change relative to the un-stimulated HUVECs are presented. Results are the mean +/− standard deviation of technical triplicates.
(TIF)

Figure S2 **ForteBio Bio Layer Interferometry traces for the anti-ENG antibody competition assay.** Wavelength shifts (WS) traces after saturation of the streptavidin biosensor with biotinylated recombinant human ENG. Comparison of the addition of the following individual anti-ENG antibodies to Endoglin alone (ENG + CTRL – bottom traces in each graph) or Endoglin saturated with the M999 antibody (top traces in each graph): (A) SN6, (B) SN6h, (C) TRC105. Average (WS) for each condition at time 290 sec and 490 sec were plotted in Figure 5B.
(TIF)

Figure S3 **ForteBio Bio Layer Interferometry traces for the BMP9 inhibition assay.** Wavelength shifts (WS) traces after saturation of the streptavidin biosensor with biotinylated recombinant human ENG. Comparison of the binding of BMP9 after saturation of the biosensor with the following individual anti-ENG antibodies: (A) CTRL IgG1k, (B) SN6, (C) SN6h, (D) M999 and (E) TRC105. Average WS for each condition at time 490 sec were plotted in figure 5D.
(TIF)

Figure S4 **FBS triggers SMAD1/5/8 phosphorylation.** HUVECs were either serum-starved for 3 hours or maintained in 10% FBS media, then stimulated with 1 ng/ml of BMP9 for 30 minutes (+) or PBS (−). Levels of pSMAD1/5/8 and SMAD1 in the total cell extracts were assessed by western blotting.
(TIF)

Figure S5 **SN6 does not affect BMP9/pSMAD1 signaling in HUVECs.** HUVECs were dispensed in each well of a 96-well plate (2500 cells/well) and incubated in serum free media for 3 hours. Antibodies were added to the cells at various concentrations 1 hour prior to stimulation with BMP9 (0.1 ng/ml) (+) or PBS (−) for 30 min. After cell lysis, levels of pSMAD1 normalized for levels of total SMAD1 were monitored using a SMAD1/pSMAD1 MSD assay. The following antibodies were used: mIgG1k (mouse IgGik isotype control), SN6 (mouse IgG1k anti-human ENG monoclonal antibody), TRC105 (human IgG1 anti-human ENG monoclonal antibody). Results are the mean +/− standard deviation of technical triplicates (Δ = P value>0.05).
(TIF)
