# Supplementary figures and images for: Endoglin Requirement for BMP9 Signaling in Endothelial Cells Reveals New Mechanism of Action for Selective Anti-Endoglin Antibodies
Source: PLoS One. 2012 Dec 27;7(12):e50920. doi: 10.1371/journal.pone.0050920 (PMC3531442; doi:10.1371/journal.pone.0050920)

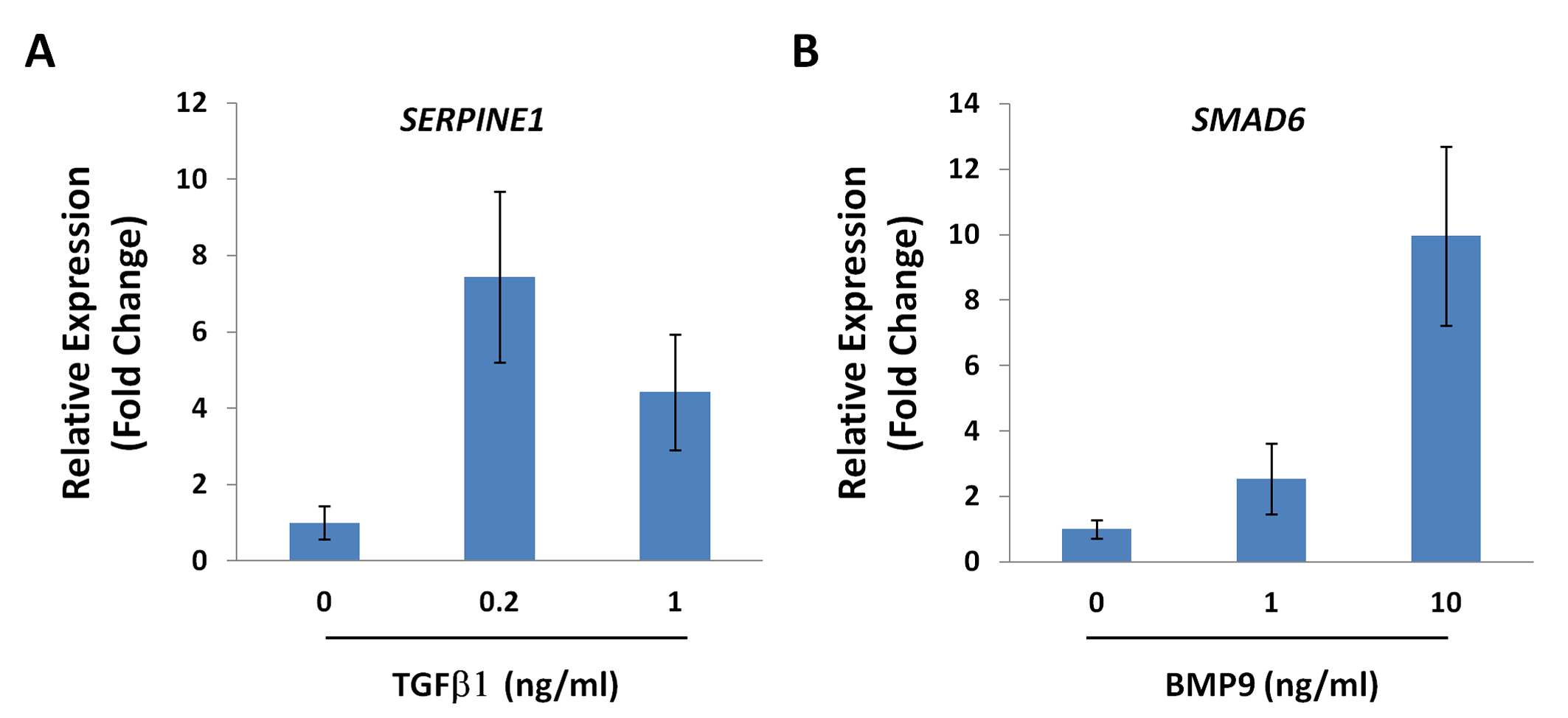

Supplement: Figure S1 — Induction of SERPINE1 and SMAD6 by TGFβ1 and BMP9, respectively. HUVECs were dispensed in each well of a 96-well plate (2500 cells/well), serum-starved overnight and stimulated with increasing amounts of recombinant BMP9 or TGFβ1. Total RNA extracts were subjected to reverse transcription and quantitative PCR assessment of the SMAD6 and SERPINE1 (A.k.a PAI-1) transcripts relative to the levels of the GAPDH transcript. Fold change relative to the un-stimulated HUVECs are presented. Results are the mean +/− standard deviation of technical triplicates. (TIF) [file pone.0050920.s001.tif]

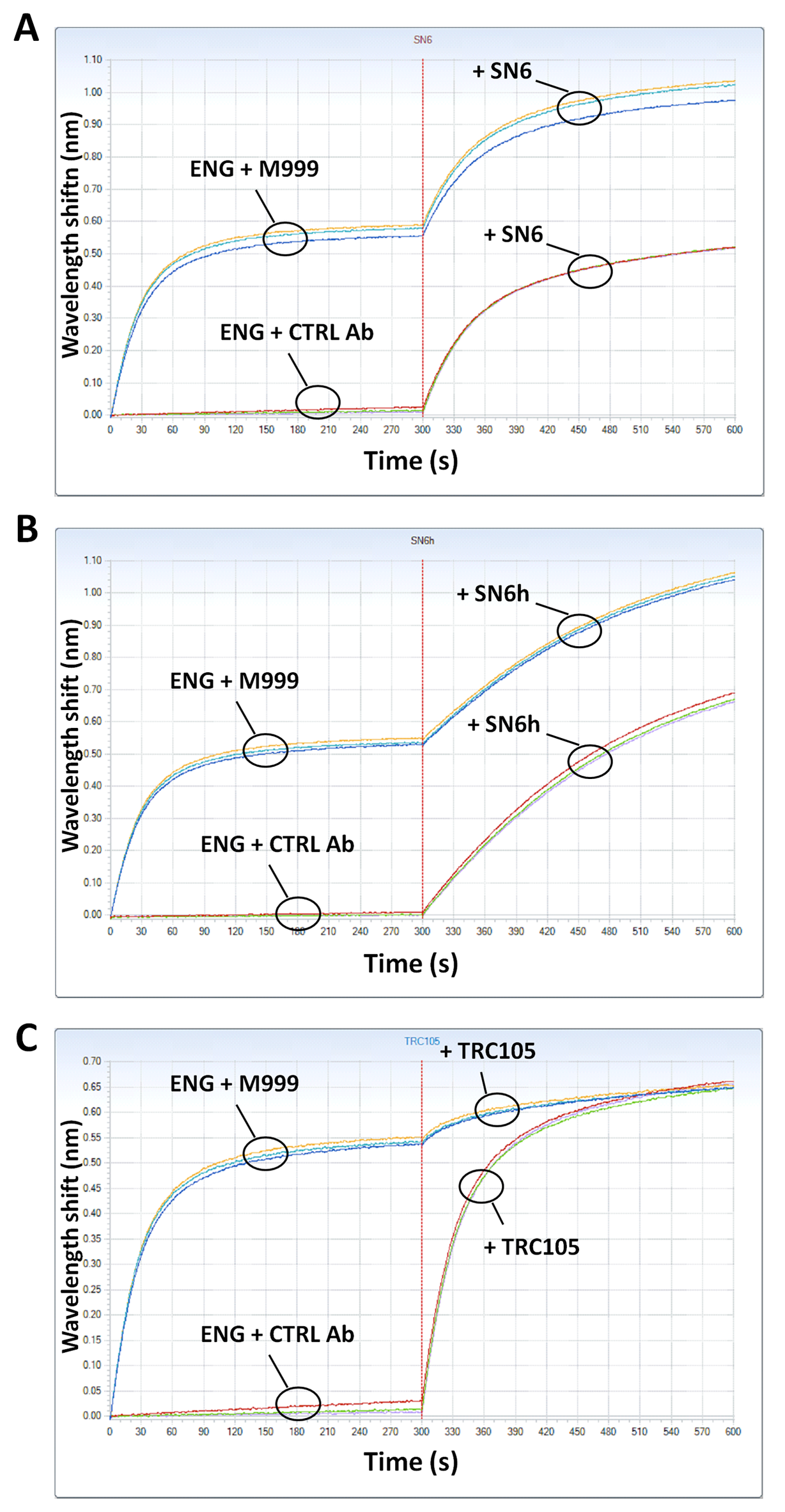

Supplement: Figure S2 — ForteBio Bio Layer Interferometry traces for the anti-ENG antibody competition assay. Wavelength shifts (WS) traces after saturation of the streptavidin biosensor with biotinylated recombinant human ENG. Comparison of the addition of the following individual anti-ENG antibodies to Endoglin alone (ENG + CTRL – bottom traces in each graph) or Endoglin saturated with the M999 antibody (top traces in each graph): (A) SN6, (B) SN6h, (C) TRC105. Average (WS) for each condition at time 290 sec and 490 sec were plotted in Figure 5B. (TIF) [file pone.0050920.s002.tif]

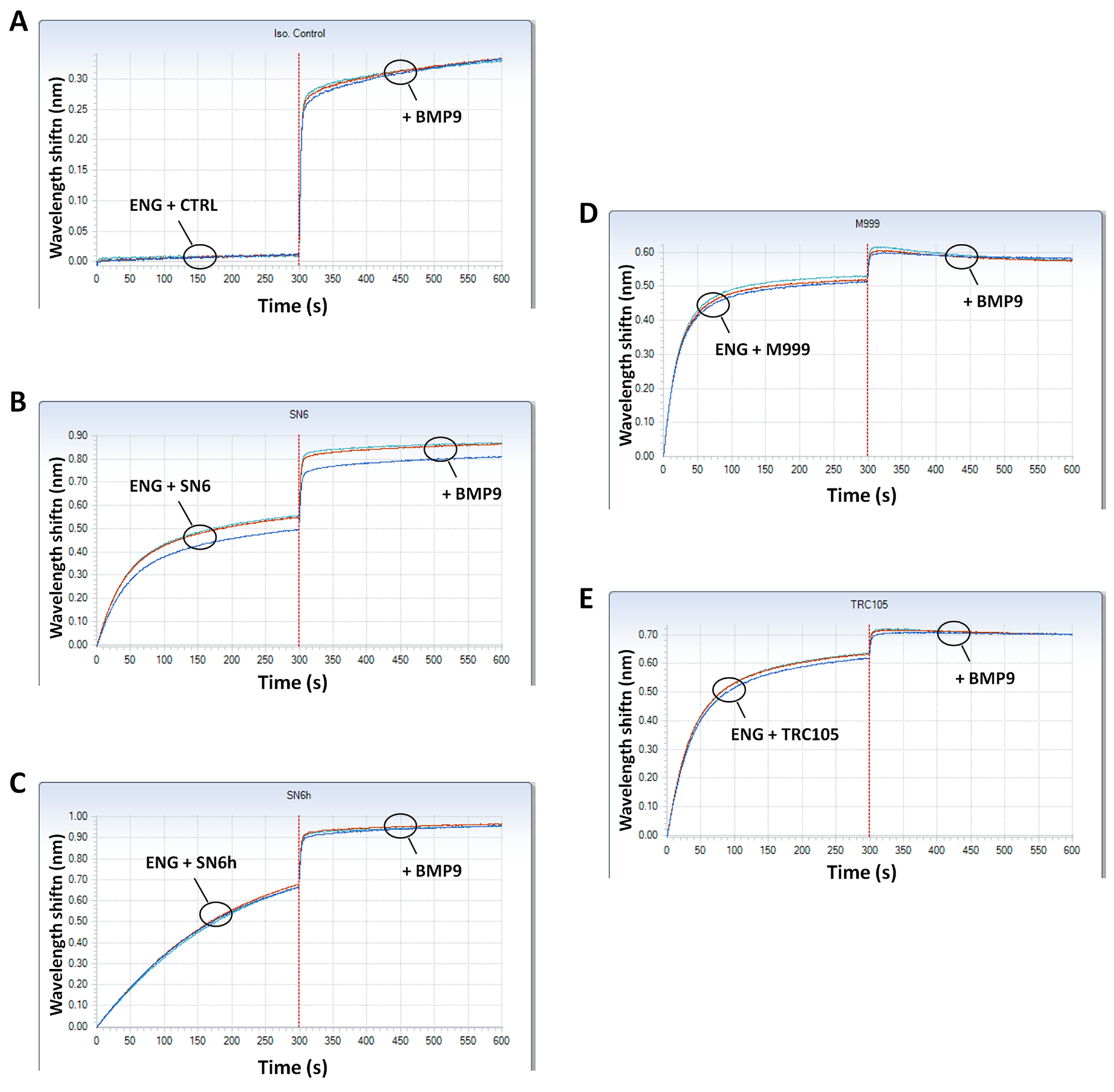

Supplement: Figure S3 — ForteBio Bio Layer Interferometry traces for the BMP9 inhibition assay. Wavelength shifts (WS) traces after saturation of the streptavidin biosensor with biotinylated recombinant human ENG. Comparison of the binding of BMP9 after saturation of the biosensor with the following individual anti-ENG antibodies: (A) CTRL IgG1k, (B) SN6, (C) SN6h, (D) M999 and (E) TRC105. Average WS for each condition at time 490 sec were plotted in figure 5D. (TIF) [file pone.0050920.s003.tif]

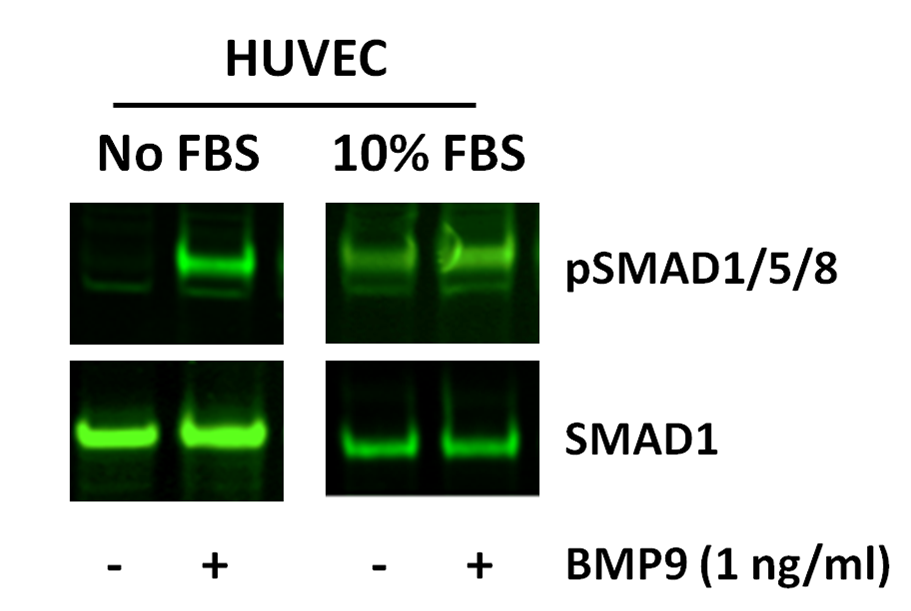

Supplement: Figure S4 — FBS triggers SMAD1/5/8 phosphorylation. HUVECs were either serum-starved for 3 hours or maintained in 10% FBS media, then stimulated with 1 ng/ml of BMP9 for 30 minutes (+) or PBS (−). Levels of pSMAD1/5/8 and SMAD1 in the total cell extracts were assessed by western blotting. (TIF) [file pone.0050920.s004.tif]

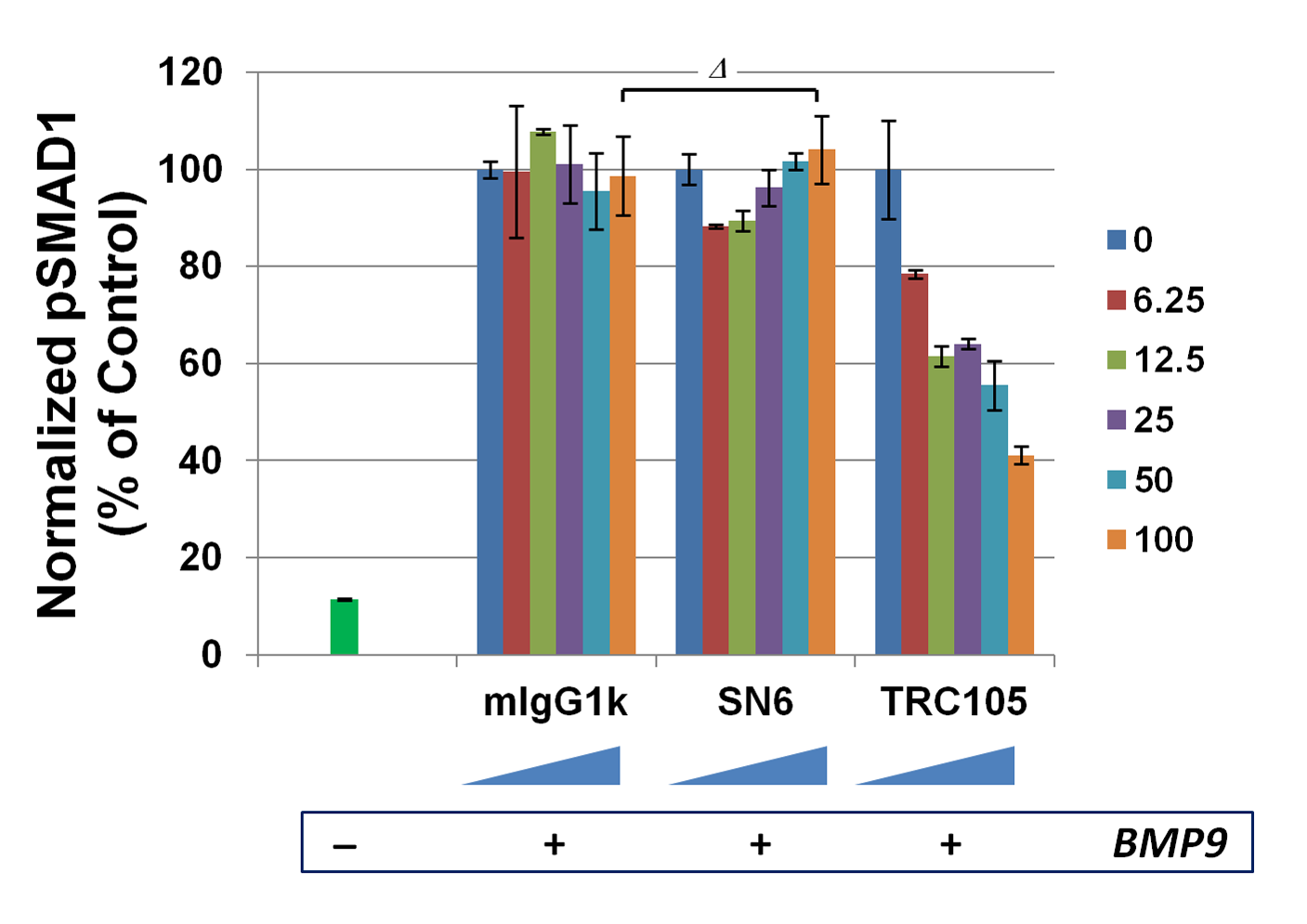

Supplement: Figure S5 — SN6 does not affect BMP9/pSMAD1 signaling in HUVECs. HUVECs were dispensed in each well of a 96-well plate (2500 cells/well) and incubated in serum free media for 3 hours. Antibodies were added to the cells at various concentrations 1 hour prior to stimulation with BMP9 (0.1 ng/ml) (+) or PBS (−) for 30 min. After cell lysis, levels of pSMAD1 normalized for levels of total SMAD1 were monitored using a SMAD1/pSMAD1 MSD assay. The following antibodies were used: mIgG1k (mouse IgGik isotype control), SN6 (mouse IgG1k anti-human ENG monoclonal antibody), TRC105 (human IgG1 anti-human ENG monoclonal antibody). Results are the mean +/− standard deviation of technical triplicates (Δ = P value>0.05). (TIF) [file pone.0050920.s005.tif]
